# Dr. Paul M. Pilcher

**Published:** 1917-02

**Authors:** 


**Dr. Paul M. Pilcher,** P. & S. 1900, died at his home in Brooklyn, Jan. 4, aged 40. He was the first editor of the Long Island Medical Journal and a surgeon of note. He is survived by his father, Dr. Lewis S. and his brother, Dr. James T. Pilcher of Brooklyn.

